# Phylogenetic lineage dynamics of global parainfluenza virus type 3 post-COVID-19 pandemic

**DOI:** 10.1128/msphere.00624-23

**Published:** 2024-03-19

**Authors:** Kihyun Lee, Kuenyoul Park, Heungsup Sung, Mi-Na Kim

**Affiliations:** 1 CJ Bioscience, Seoul, South Korea; 2 Department of Laboratory Medicine, Sanggye Paik Hospital, School of Medicine, Inje University, Seoul, South Korea; 3 Department of Laboratory Medicine, Asan Medical Center, University of Ulsan College of Medicine, Seoul, South Korea; University of Michigan, Ann Arbor, Michigan, USA

**Keywords:** parainfluenza virus, molecular epidemiology, COVID-19

## Abstract

During the coronavirus disease 2019 (COVID-19) pandemic, outbreaks of parainfluenza virus type 3 (PIV-3) decreased due to infection control measures. However, a post-pandemic resurgence of PIV-3 has recently been observed. Nonetheless, the role of viral genetic epidemiology, possibly influenced by a genetic bottleneck effect, remains unexplored. We investigated the phylogenetic structure of the publicly available PIV-3 whole-genome and hemagglutinin-neuraminidase (HN) gene sequences spanning the last 65 years, including the COVID-19 pandemic. Sequences were retrieved from the nucleotide database of the National Center for Biotechnology Information using the search term “Human respirovirus 3.” Sequence subsets covering all six genes of PIV-3 or the HN gene were designated as the whole-genome and HN surveillance data sets, respectively. Using these data sets, we constructed maximum-likelihood phylogenetic trees and performed a time-scaled analysis using a Bayesian SkyGrid coalescent prior. A total of 455 whole-genome and 1,139 HN gene sequences were extracted, revealing 10 and 11 distinct lineages, respectively, with >98% concurrence in lineage assignments. During the 2020 COVID-19 pandemic, only three single-lineage clusters were identified in Japan, Korea, and the USA. The inferred year of origin for PIV-3 was 1938 (1903–1963) for the whole-genome data set and 1955 (1930–1963) for the HN gene data set. Our study suggests that PIV-3 epidemics in the post-COVID era are likely influenced by a pandemic-driven bottleneck phenomenon and supports previous hypotheses suggesting s that PIV-3 originated during the early half of the 20th century.

**IMPORTANCE:**

Using publicly available parainfluenza virus type 3 (PIV-3) whole-genome sequences, we estimated that PIV-3 originated during the 1930s, consistent with previous hypotheses. Lineage typing and time-scaled phylogenetic analysis revealed that PIV-3 experienced a bottleneck phenomenon in Korea and the USA during the coronavirus disease 2019 pandemic. We identified the conservative hemagglutinin-neuraminidase gene as a viable alternative marker in long-term epidemiological studies of PIV-3 when whole-genome analysis is limited.

## OBSERVATION

The parainfluenza virus (PIV) is responsible for up to 13% of acute lower respiratory tract infections (1). *Respirovirus pneumoniae*, conventionally known as PIV type 3 (PIV-3), is the most prevalent species of PIV (2). Enhanced infection control measures implemented during the coronavirus disease 2019 (COVID-19) pandemic dramatically reduced viral respiratory infections (3). However, subsequent easing of these measures has been accompanied by outbreaks of the enveloped PIV-3 and respiratory syncytial virus (3, 4). The COVID-19 pandemic greatly affected the genomic diversity of influenza viruses (5) and potentially other enveloped viruses. However, the changes in the molecular epidemiology of PIV-3 before and during the COVID-19 pandemic remain unclear. Among the six genes of PIV-3, the hemagglutinin-neuraminidase (HN) gene is frequently used in epidemiology (6–8). Consequently, this study investigated the changes in the molecular epidemiology of PIV-3 during the COVID-19 pandemic by analyzing publicly available whole-genome and HN gene sequence data for the last 65 years.

We screened 2,878 PIV-3 sequences from GenBank and identified 455 nearly complete genomes, along with 1,139 nearly full-length HN gene sequences (Table S1). Data on the country of origin and collection year were available for 447 whole-genome and 1,108 HN gene records. Only six whole-genome and 18 HN gene records were generated from samples collected after 2020 (Table S2).

**Fig 1:**
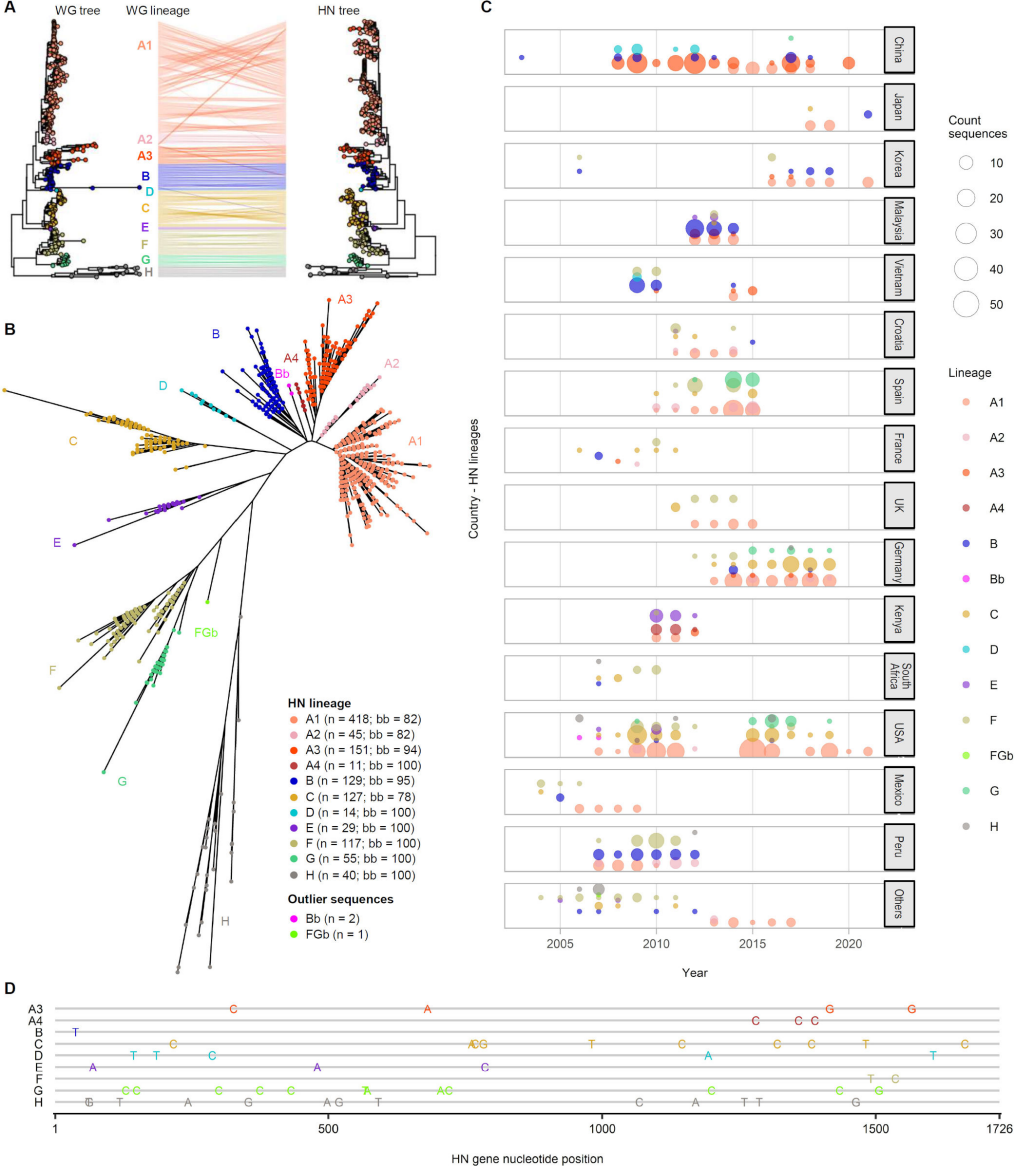
Phylogenetic analysis of publicly available PIV-3 sequences. (**A**) Comparison of maximum-likelihood phylogenetic trees reconstructed from whole-genome (left, *N* = 455; 10 lineages) and HN gene sequences (right, *N* = 1,139; 11 lineages). The tips of both trees are color-coded according to lineage assignments based on the whole-genome tree, and lines connect the same samples in the two trees. (**B**) Lineage assignment based on HN gene sequences. Bb and FGb are defined as basal outlier groups rather than lineages because of their small sizes. (**C**) Chronological distribution of PIV-3 based on HN lineage per country (2003–2021). (**D**) Distribution of signature variants characterizing HN lineages.

Phylogenetic analysis was conducted on PIV-3 sequences using both the whole-genome and HN gene data sets. No recombination signal was detected in the HN gene, whereas it was detected in three 1,000 bp regions inside the M, F, and L genes (Table S3) (9, 10). Regions impacted by recombination were removed from the whole-genome alignment. The normalized similarities between the maximum-likelihood phylogenetic trees based on the whole-genome and HN data sets were 0.712 and 0.614, respectively, using Nye’s method and the generalized Robinson-Foulds metric (Fig. 1A). Based on the phylogenetic trees, we identified 10 distinct lineages in the whole-genome data set and 11 lineages and two basal outlier sequences in the HN gene data set (Bb, *n* = 2; and FGb, *n* = 1; Tables S4 and S5).

Branch supports for the defined lineages were ≥78% in all cases based on the ultrafast bootstrap score, except for the basal outlier labels in the HN tree ending with “b” (Fig. 1B). Lineage assignments based on both data sets were consistent: among the 455 viral samples in both the whole-genome and HN data sets, the lineage assignments were coherent in 448 cases (98.5%) and disparate in seven cases (1.5%) (Fig. 1A), among which four cases were between A1 and A3, and the other three were more distantly mismatched. The three distantly mismatched sequences were either recombinant sequences (EU326526.1 and FJ455842.2, A3 by whole genome and B by HN) (11) or the outlier identified in the temporal phylogenetic signal analysis (KF687357.1, B by whole genome and C by HN). Retrospective analysis of the whole genome confirmed the placement of the FGb outlier sequence (KF530237.1), which was excluded from the whole-genome tree due to low coverage over the L gene, and branching out from the ancestral lineage to the F and G clades (Fig. S1), as suggested by the HN gene phylogeny.

**Fig 2:**
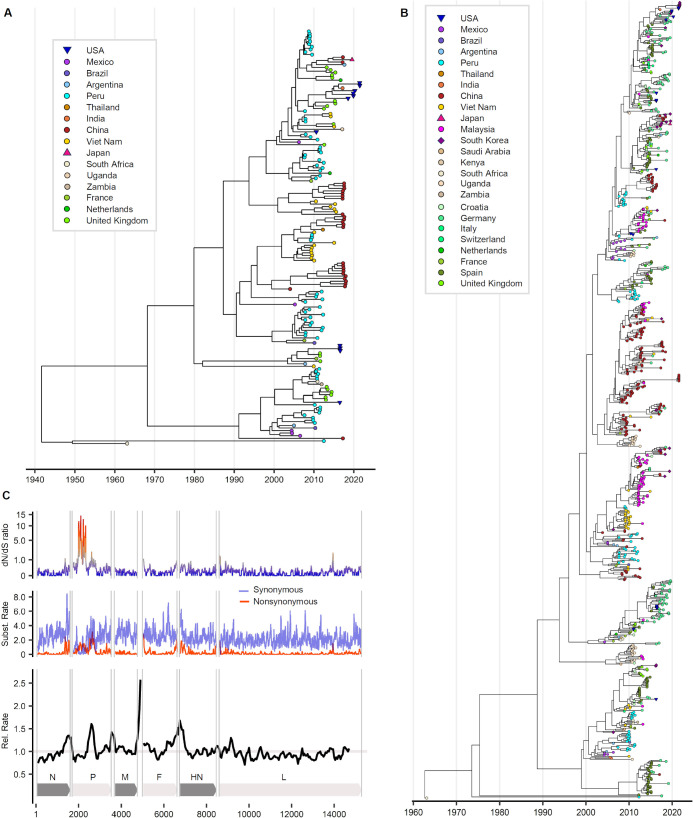
Time-scaled evolutionary analysis of PIV-3. (**A**) Maximum clade credibility (MCC) tree of whole-genome sequences for which sample collection dates were available (*N* = 161). (**B**) MCC tree of HN gene sequences (*N* = 793). Outlier taxa were excluded following root-to-tip regression analysis. (**C**) Variation in substitution rates across the whole genome of PIV-3.

During the 2020 COVID-19 pandemic (2020–present), only three clusters were identified in Japan, Korea, and the USA; the PIV-3 sequences from Korea and the USA belonged to the same lineage (Fig. 1C). Signature variants characteristic of the HN lineage were distributed throughout the HN gene (Fig. 1D). Time-scale phylogenetic analysis revealed the relatively close proximity of sequences from the same country. PIV-3 sequences from Korea and the USA showed similarity in HN gene time-scale phylogeny. The mean inferred year of origin (time to most recent common ancestor; tMRCA) for PIV-3 was 1938 (95% highest posterior density; 95% HPD 1903–1963) for the whole-genome data set and 1955 (95% HPD 1930–1963) for the HN gene data set (Fig. 2A and B). The whole-genome data revealed a relatively low evolutionary rate in the HN gene region (Fig. 2C).

To enable efficient global genetic surveillance, we propose an 11-lineage classification scheme based on HN gene sequences, which builds on a previously proposed scheme (12) using a larger and more diverse data set (Table S6) and demonstrates good compatibility with whole genome-based phylogenetic lineages. Although most genomes were sequenced during the 2010s, there was a notable decrease in the availability of PIV-3 genomes in the early 2020s (corresponding to <2% of the PIV-3 genomes reported in this study). This sharp decline in sequencing data may reflect a decrease in PIV-3 infections related to COVID-19 pandemic regulations (3, 13) or a shift in genetic surveillance efforts toward the coronavirus, among other reasons. Only one lineage persisted in Korea and the USA during the pandemic, while a new lineage emerged in Japan during the pandemic. However, due to the severe lack of sequence data during the pandemic, it remains challenging to elucidate the impact of COVID-19 on PIV-3 lineage dynamics. With the World Health Organization declaring an end to the COVID-19 pandemic (14), PIV-3 transmission is expected to return to pre-pandemic levels. However, it remains challenging to predict how the genetic diversity of PIV-3 will change from its past to post-COVID epidemics. Observations in other respiratory viruses (e.g., respiratory syncytial viruses) (15) suggested that reduced incidence during COVID-19 may have led to genetic bottleneck events, highlighting the importance of continued molecular surveillance for elucidating the influence of pandemics on viral evolution and transmission. Therefore, molecular surveillance of PIV-3 using our lineage scheme could help track changes in the dominant clone and divergence of PIV-3. Phylogenies based on the HN gene and whole genome were congruent, likely owing to the conservative nature of the HN gene, which is preferred for long-term and global molecular epidemiology (16). The relatively low evolutionary rate in the HN gene was consistent with its conserved nature (17). Therefore, the HN gene represents a suitable alternative for investigating the molecular epidemiology of PIV-3, particularly in long-term studies.

The time at which PIV-3 originated remains controversial, as shown in the comparison of tMRCA in our and previous studies (Table S7). Between the different studies, we observed substantial variation in, for example, the number and temporal distribution of samples, target locus, treatment of recombination, substitution and clock models, and selection of coalescent models. Notably, the HN-based tMRCA was more recent than the whole genome-based tMRCA. The whole-genome analysis also indicated a later origin (1938) than that reported in previous studies (1905–1920) (11, 17). Although our HN- and whole genome-based tMRCA estimates were both later than the previously reported HN- and whole genome-based tMRCA estimates, respectively (11, 18), the previous tMRCA values were all within the 95% highest probability density range in our analyses. The differences in the estimated year of origin based on whole-genome data may simply stem from variations in the methods used across studies. Nevertheless, our study supports previous hypotheses on the origin of PIV-3 dating back to the early half of the 20th century.

Since PIV-3 transmission was dramatically reduced during the pandemic, our study was limited by the small sample size of post-pandemic sequence data. Therefore, a follow-up study using a larger number of post-COVID-19 whole-genome or HN gene sequences is needed to fully elucidate the changes in the molecular epidemiology of PIV-3. Second, our phylogenetic lineage-based framework used the HN gene only. The HN gene is an important marker in molecular epidemiological studies of PIV-3 and is expected to be a useful marker for epidemiological surveillance because of its relatively slow evolutionary rate. However, it may not be ideal for short-term outbreak investigations, where the use of a hypervariable region would be more appropriate (17).

In conclusion, our estimated origin of PIV-3 in the 1930s is consistent with previous hypotheses. During the COVID-19 pandemic, PIV-3 experienced a bottleneck phenomenon in Korea and the USA, as observed through lineage typing and time-scaled phylogenetic analysis. Our study underscores the suitability of the conserved HN gene in long-term epidemiological studies on PIV-3, especially when whole-genome analysis is limited.

## MATERIALS AND METHODS

A comprehensive description of the Materials and Methods is provided in the Supplementary Material. Briefly, we retrieved the PIV-3 sequence accession numbers from the National Center for Biotechnology Information nucleotide database using the search term “Human respirovirus 3” and extracted sequences and source metadata (Table S1). Sequence subsets covering all six genes of PIV-3 and HN and showing >90% alignment coverage through BLASTN were designated as the whole-genome and HN surveillance data sets, respectively. We constructed maximum-likelihood phylogenetic trees from these alignments using IQ-Tree 2.0.3 (19), checked for temporal signals using TempEst 1.5.3 (20), and removed outliers. Time-calibrated phylogenetic analysis was performed on both data sets using a Bayesian SkyGrid coalescent prior (21). Synonymous and non-synonymous substitution rates were inferred from the alignment positions using the single likelihood ancestor counting (SLAC) method in Datamonkey (22). The protocol for PIV-3 lineage typing is described on GitHub (doi.org/10.5281/zenodo.10695952).

## Data Availability

Sequence accession numbers and the associated metadata are provided in Table S1. The scripts and input files are provided on GitHub [https://github.com/kihyunee/parainfluenza_lineages].

## SUPPLEMENTAL MATERIAL

Supplemental materials msphere.00624-23-s0001.docx Material and methods, legends for Tables S1-S6, Table S7, and Figure S1.

Supplemental tables msphere.00624-23-s0002.xlsx Tables S1 to S6.

ASM does not own the copyrights to Supplemental Material that may be linked to, or accessed through, an article. The authors have granted ASM a non-exclusive, world-wide license to publish the Supplemental Material files. Please contact the corresponding author directly for reuse.
