# Supplementary material for: Phylogenetic lineage dynamics of global parainfluenza virus type 3 post-COVID-19 pandemic
Source: mSphere. 2024 Mar 19;9(4):e00624-23. doi: 10.1128/msphere.00624-23 (PMC11036794; doi:10.1128/msphere.00624-23)
Supplement: Supplemental materials — Material and methods, legends for Tables S1-S6, Table S7, and Figure S1. [file msphere.00624-23-s0001.docx]

**Supplementary Material for**

**Phylogenetic Lineage Dynamics of Global Parainfluenza Virus Type 3 Post-COVID-19 Pandemic**

**Kihyun Lee^a^, Kuenyoul Park^b#^, Heungsup Sung^c#^ and Mi-Na Kim^c^**

^a^CJ Bioscience, Seoul, Korea.

^b^Department of Laboratory Medicine, Sanggye Paik Hospital, School of Medicine, Inje University, Seoul, Korea

^c^Department of Laboratory Medicine, Asan Medical Center, University of Ulsan College of Medicine, Seoul, Korea

^#^Address correspondence to Kuenyoul Park, kuenyoul.park@gmail.com and Heungsup Sung, sung@amc.seoul.kr.

**Materials and Methods**

***PIV-3 reference genomes and CDSs***

From the NCBI’s collection of complete viral genome sequences, we identified accessions with a “taxon_name” field matching “Human parainfluenza virus 3". From the retrieved complete genome sequences (n = 185), we combined the CDS sequences of the annotated protein-coding genes (n = 1,202), and dereplicated the CDS sequences at 99% identity using cd-hit-est 4.8.1. The resulting non-redundant CDS collection comprised the genes for six proteins: the nucleocapsid protein (NP), phosphoprotein (P; encompassing the regions for C protein and D protein), matrix protein (M), hemagglutinin neuraminidase (HN), fusion protein (F), and RNA-dependent RNA polymerase (L). These reference CDS sequences were utilized subsequently to assess the completeness of the GenBank entries in the surveillance dataset.

***Sequence data collection***

We searched for accession numbers of GenBank records labeled as parainfluenza virus type 3 by using the query term "Human respirovirus 3"[porgn:__txid11216] on the NCBI GenBank. The search was conducted on April 23, 2023, and we retrieved 2,842 accession numbers. We downloaded the FASTA and GenBank (.gb) files for all of these accessions and used our custom script to parse the source metadata from the GenBank records, extracting information about the geographic location and date of sample collection.

To refine the surveillance datasets based on the whole genome (WG) and HN gene sequences, we assessed the coverage of each sequence entry across the six known CDSs of PIV-3 and the HN gene, respectively. We performed blastn alignment by querying each nucleotide sequence against the non-redundant reference CDS sequences. Subsequently, we calculated the coverage of each nucleotide sequence over each of the six CDS regions. Sequence entries with over 90% coverage breadth across all six CDS regions were designated as valid WG sequences, while those with over 90% coverage breadth to the HN gene were designated as valid HN sequences.

We further stratified the surveillance datasets further by metadata availability for collection date and geographic location. We omitted sequences without isolation date (year and month) or country of origin from the time-scaled phylogenetic and phylodynamic analysis. Nonetheless, we incorporated these excluded sequences in the phylogenetic reconstruction to define viral lineages. For sequences that include date information in the form of the collection month, we used the first day of the given month as pseudo sampling date to prepare the input for time-calibrated phylogenetic analyses.

***Multiple sequence alignments***

We constructed whole genome multiple sequence alignment by first aligning each sequence in the WG surveillance dataset against a fixed reference sequence (KF530231) using the G-INS-i algorithm implemented in MAFFT version 7.520. We then merged the columns covered by at least 90% of the entries. The multiple sequence alignments derived from this process were used in all WG-based phylogenetic analyses. For the HN gene sequences, we generated multiple sequence alignments using Muscle version 5.1 with default parameters. We utilized the resulting HN gene sequence alignment for phylogenetic lineage analysis based on the HN gene. We also produced codon-level multiple sequence alignments of the coding sequences of each of the six CDSs by initially building amino acid alignments using Muscle5 and then translating the amino acids back into the codon-by-codon nucleotide alignments. The codon-level alignments were utilized in the time scale phylogenetic analysis and the SLAC analysis.

***Phylogenetic analyses***

Recombination was tested using PhiPack version 1.1-4 and 3SEQ version 1.8.0 with default parameters. For gene-level tests, we subjected single-gene alignments to the PhiPack test and determined the presence of recombination based on a cutoff of P < 0.05. We generated non-overlapping 1,000-bp windows from the whole-genome multiple sequence alignment, generating 15 windows with 1,000 columns and a 16th window with 369 columns. Windows corresponding to P < 0.05 based on the PhiPack test were removed from the initial whole-genome alignment matrix to yield a 13,000-bp alignment. This putatively recombination-free alignment was used in the downstream whole-genome maximum likelihood phylogenetic tree reconstruction and time tree analysis.

We used IQ-Tree version 2.0.3 to select the optimal substitution model and reconstruct the maximum-likelihood phylogenetic tree. For whole-genome alignment with recombination sites removed, GTR+F+R3 was selected as the optimal substitution model. For HN-gene alignment, TIM+F+R4 was selected as the optimal model. The resulting maximum likelihood trees were used for lineage classification.

For time-calibrated phylogenetic analyses of WG and HN gene sequences, we initially assessed the temporal signal in each dataset using TempEst version 1.5.3 with the maximum-likelihood trees as the input trees. Following manual evaluation of the TempEst root-to-tip regression results, we eliminated the outlier sequences (sequences whose absolute value of the residual was larger than 0.01; n = 5 from WG, and n = 8 from HN gene; list of outliers can be found in Supplementary Table 1) prior to the subsequent coalescent analysis. Next, we conducted coalescent analyses on both the WG and the HN gene datasets using the Bayesian Skygrid prior. We utilized the rooted trees generated using TempEst as the starting tree for each corresponding dataset and applied a relaxed clock model to accommodate the rate variation across branches, considering that the sequence data span diverse lineages and 60 years. We used the HKY substitution model with four rate categories and set the prior mean value for the substitution rate at 0.001. The number of rate categories was selected based on the IQ-Tree ModelFinder results, and the starting mean value for the substitution rate was based on the TempEst estimate of the whole-genome substitution rate (0.00055 and 0.00049 for the whole-genome and HN datasets, respectively). We ran the MCMC chain three times, giving 40,000,000 generation for each chain, and generated maximum clade credibility trees from the output trees.

We run the single-likelihood ancestor counting (SLAC) analysis provided in the datamonkey server (https://www.datamonkey.org/slac) to determine the evolutionary rates at each position of the codon-level multiple sequence alignments for the six genes including the NP, P, M, HN, F, and L genes.

***Lineage assignments***

We manually examined the maximum-likelihood phylogenetic trees of the WG and the HN gene sequences, selecting monophyletic branches that were well-supported (ultrafast bootstrap support > 0.75) and clearly separated from one another (based on manual judgement). We plotted time series of PIV3 virus HN lineage distributions from 2003 to 2021 for the countries with at least 10 sequences, using the HN gene sequences that include both collection year (allowing the absence of month and date information) and country-of-origin.

The sequence data and in-house scripts used in the phylogenetic analyses are available in the following DOI: 10.5281/zenodo.10695952.

**Supplementary Tables**

The tables S1 – S6 can be found in a separate spreadsheet (excel) file providing each of the following tables as a separate tab.

**Table S1. Accession numbers and source metadata of the PIV-3 sequences analyzed in this study**

**Table S2. Distribution of the number of collected sequence data by year and country**

**Table S3. Recombination test across the genomic regions of PIV-3**

**Table S4. Lineage assignments based on the WG and the HN gene sequences**

**Table S5. Intra- and inter-lineage divergence of sequences**

**Table S6. Comparison with a previously published HN gene-based classification**

**Table S7. Estimated time to most recent common ancestors of PIV-3**

|  | **Linster et al. 2018** | **Bose et al. 2019** | **Smielewska et al. 2018** | | **This study** | |
| --- | --- | --- | --- | --- | --- | --- |
| **Locus used** | **HN** | **Whole genome** | **Hypervariable region between M and F** | **Whole genome** | **HN** | **Whole genome** |
| **tMRCA (95% HPD)** | 1943 (1938–1948) | ca. 1905  (not explicitly stated) | ca. 1920  (not explicitly stated) | NA | 1955 (1930 – 1963) | 1938 (1903 – 1963) |
| **Substitution rate**  **(95% HPD) (/site/year)** | 8.4E-4 (7.7E-4 – 9.1E-4) | 3.6E-4 (3.3E-4 – 3.9E-4) | 1E-3 | 4.2E-4 | 1.1E-3 (6.8E-4 – 1.4E-3) | 6.7E-4 (5.1E-4 – 8.3E-4) |
| **Number of sequences** | 519 | 268 | 56 | 56 | 793 | 161 |
| **Sampled dates** | 1957 – 2015 | 2004 – 2016 | 2004 – 2017 | 2004 – 2017 | 1963 – 2023 | 1963 – 2023 |
| **Software** | BEAST v1.8.3 | BEAST v1.8.2 | BEAST v1.8.4 | BEAST v1.8.4 | BEAST v1.10.4 | BEAST v1.10.4 |
| **Substitution model used** | HKY | HKY + I + G4 | TRN +I | GTR + I + G | HKY + G4 | HKY + G4 |
| **Clock model used** | Strict clock | Strict clock | Strict clock | Uncorrelated relaxed clock | Uncorrelated relaxed clock | Uncorrelated relaxed clock |
| **Tree prior used** | Constant coalescent | Piecewise-constant Bayesian Skyline | Constant coalescent | NA | Bayesian Skygrid | Bayesian Skygrid |
| **MCMC length * runs** | 100 million | 100 million * 2 – 5 | 10 million | 600 million | 40 million * 3 | 40 million * 3 |
| **Recombination treatment** | Not treated | Recombined sequences removed | Not treated | Not treated | Recombined sites removed | Recombined sites removed |


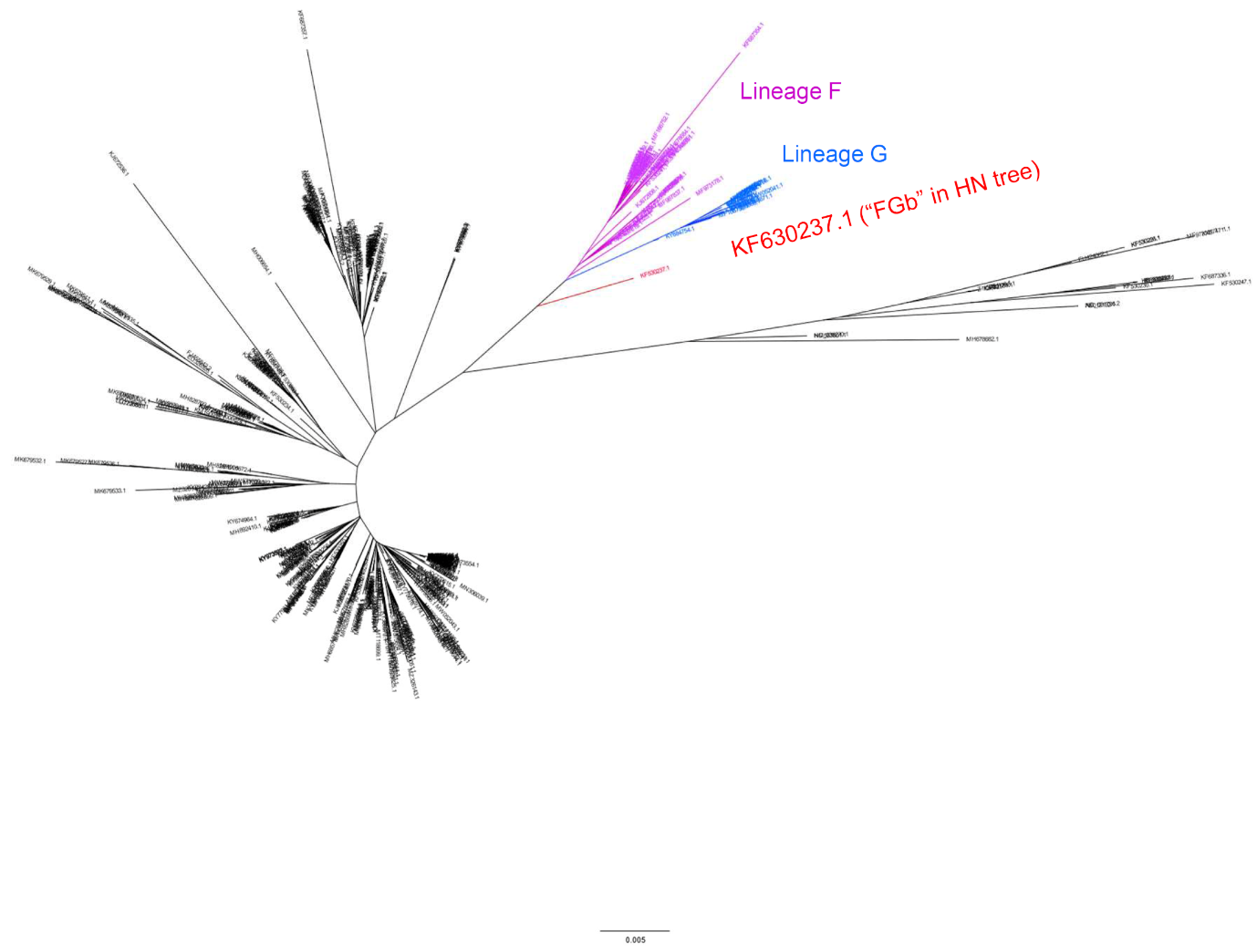


**Figure S1. Whole genome phylogenetic analysis of the basal outlier group FGb**

The sequence assigned as a basal outlier group FGb (KF530237.1) based on hemagglutinin-neuraminidase (HN) gene phylogeny was re-analyzed with whole genome (WG) phylogeny here. We added the sequence KF530237.1 to the previously constructed WG alignment containing 455 sequences. Following the main WG phylogenetic analysis, we removed the regions in the whole genome alignment that showed signals of recombination and ran IQ-Tree 2 with GTR+F+R3 substitution model. The clades corresponding to the lineages F and G are indicated by distinctive colors.
